# Cancer education in Lithuania

**DOI:** 10.3332/ecancer.2014.487

**Published:** 2014-12-04

**Authors:** Ramune Mineikyte, Ernestas Janulionis, Jurgita Liutkeviciute-Navickiene, Vydmantas Atkocius

**Affiliations:** National Cancer Institute, Santariskiu Street 1, 08660 Vilnius, Lithuania

**Keywords:** cancer education, Lithuania, oncological educational programmes

## Abstract

The aim of this article is to describe cancer education in Lithuania according to the data of 2013.

In Lithuania, there are the following stages of education for physicians: basic education through integrated studies of medicine (six years), postgraduate education through residency studies (four to five years), and continuing professional development.

In recent years, integrated studies of medicine have been the most popular specialty. Oncology is incorporated into the teaching courses in medicine programmes. In each university, an oncology course is mandatory during these studies.

In Lithuania, there are two types of specialists related to oncology: medical oncologists and radiation oncologists. These oncologists complete multidisciplinary residency study programmes in the clinics.

To receive a doctoral degree, specialists may join PhD programmes at any of the accredited universities. In recent years the number of dissertations in oncology has grown. Notably, oncology is chosen not only by students in the field of medicine. It also becomes the choice of those seeking a doctorate in the fields of nursing, public health, biochemistry, and physics.

The professional development of oncologists is a lifelong commitment. In Lithuania, continuing specialist medical training is mandatory. This requirement is ensured with the process of licensing of medical practice.

All Lithuanian study programmes are certificated by an independent public agency and are recognised by a number of other countries as well.

## Introduction

**Figure 1.**
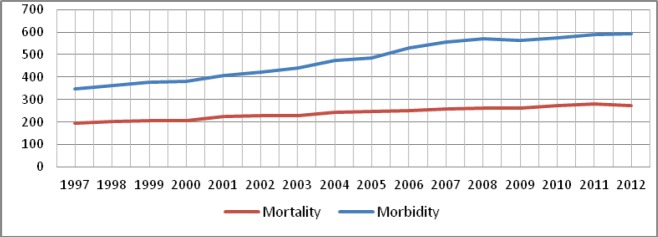
Morbidity and mortality caused by cancer per 100,000 population, Lithuania.

The purpose of the education and the training of health-care professionals is to increase workforce capacity and decrease the burden of cancer. In Lithuania, the number of new cancer patients per 100,000 people has increased twice during the last two decades [1]. In 2012, the mortality from tumours was 19.8% of all deaths, and since 2000, the mortality has increased by 31.5% (Figure 1) [2].

Cancer patients are often treated by a multidisciplinary team of oncologists. This approach is used because cancer treatment frequently involves a combination of surgery, chemotherapy, and radiation therapy. Of note is the fact that in Lithuania there are only two independent specialities in oncology: medical oncology and radiation oncology. But the process of diagnosing and treating cancer is complex and often involves a team of specialists: surgeons, radiologists, medical physicists, pathologists, palliative care experts, psychologists, nurses, and other health-care team members. So, in order to assure the quality of cancer treatment, Lithuania has to educate and train highly qualified health-care personnel. Medical professionals in Lithuania are trained in ten schools: four universities and six colleges.

In Lithuania at the end of 2012 there were only 0.4 practicing oncologists per 10,000 living in the country regions [3]. The estimates for a future demand for physicians’ numbers into 2025 shows that the demand shall increase for all physicians [4]. Besides the lack of qualified physicians, another huge issue in the health care of Lithuania is migration. Research shows that in the period of 2004–2010 about 3% of health-care specialists emigrated abroad. Another problem is an increase in the median age of health-care specialists. Forecasts are that 40–60% of currently employed medical specialists will leave their positions by the year 2025 because of ageing [2]. It will result in their replacement by newly trained specialists, also in the field of oncology.

In this article, the main focus of attention is the education and training of medical specialists in oncology. As the World Federation for Medical Education proposes in its Global Standards for Quality Improvement in Medical Education [5], in Lithuania correspondingly there are three consecutive stages of a physician’s education: a) basic medical education (six years); b) postgraduate medical education (four to five years); and c) continuing professional development (CPD).

## Methods

The databases (Lithuanian Cancer Registry (1992–2011), Health Information Centre, Department of Statistics), curriculums of the medical studies, internal universities documents, global standards for medical education, national (legal acts, medical norms, resolutions of the Government of the Republic of Lithuania, orders of the Minister of Health and of the Minister of Education and Science, etc.) and international legislation, which regulates medical education processes, were all analysed.

The data from the websites of different universities were also used, and a literature analysis was carried out.

An inclusive and detailed presentation of postgraduate cancer education in Lithuania was published in *Journal of Cancer Education* [6], and it is for this reason that postgraduate education is described minimally in this article.

## Results

### General arrangement

Two Universities in Lithuania are the sites for education and training in medicine: Vilnius University (VU) and Lithuanian University of Health Sciences (LUHS). In Lithuania, only integrated (combining the Bachelor’s and Master’s degrees) studies of medicine are carried out. The course takes six years, including a one-year internship. Up until 2008–2009, graduates of medicine faculties gained their clinical experience separately, that is, during the period of internship organised according to the principles of residency. Since 2009–2010, after the legal basis in the Republic of Lithuania was altered, internship has become a part of the programme of integrated studies of medicine. It is now as a module of clinical medical practice (internship).

After completion of the basic medical qualification, physicians develop competencies in the postgraduate medical education and training (third stage of university higher education studies), which in Lithuania are called ‘residency studies’. All the oncology trainees complete rigorous residency training in the clinics. In 2013, there were 18 residents from the medical oncology programme and 15 residents from the radiation oncology programme who studied in two Lithuanian universities.

The third level of medicine education is doctoral (PhD) studies. PhD studies are jointly organised by higher education and research institutions. The duration of regular PhD studies is up to four years, extended studies last up to six years.

To ensure optimal treatment and care for cancer patients, oncologists participate in continuing medical education (CME) and continuing professional development (CPD) programmes. CME in Lithuanian law is defined as an education for acquiring additional knowledge and skills in the medical field, and also obtaining professional qualifications in a narrow area of medical practice. In our state, as in the majority of East and Central European countries, CME, to raise the professional qualifications of medical specialists, is mandatory. This requirement is ensured with the process of the licensing of medical practice. Recertification is carried every five years. The CME system in Lithuania is based on an hour system. Since 2007, obligatory hours of professional medical training for doctors decreased from 200 to 120 hours per five year period.

### Admission

To apply to the undergraduate medical programme, the student must hold a secondary or equivalent education certificate. Admission is arranged competitively and is based on the ratings of secondary education scores. VU admits about 200 and LUHS about 300 students in their medical programmes per year. The popularity of the field is very high—in recent years, medical studies have always been in the first place in the top ten of the most popular specialties.

**Figure 2.**
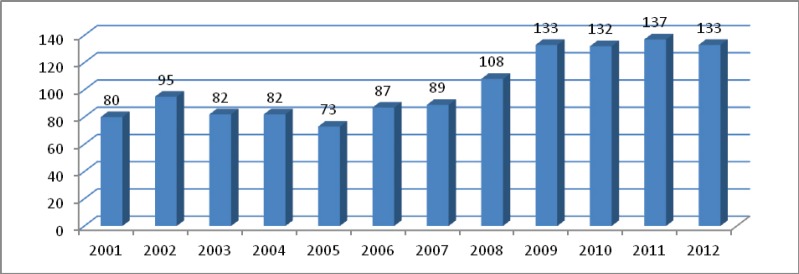
Graduates of medicine studies at universities in Lithuania (2001–2012), per million population.

According to the data of the Lithuanian Department of Statistics [7], in recent years (2009–2012) the number of graduates in university medical studies has remained stable (Figure 2). A decade ago, the figure was significantly lower, and this still has a negative effect on health care, with a lack of medical specialists, and the oncologists being among them.

An applicant to residency studies must have a professional qualification of being a medical physician, a diploma of higher education, and a certificate of internship, accredited by the law of the Republic of Lithuania. Residency is granted via a competition. The grade of the residency contest is composed of the median grade from all assessed marks throughout the programme studies, the final exam grade, and the median grade of all affiliate disciplines of medical studies (Table 1). Also an assessment of clinical medical practice (only in LUHS), and student achievements in the medical field are added to the residency contest evaluation. For those applying for the Medical and Radiation Oncology residency there is an obligatory motivational interview. Every year, 6–8 residents are accepted into the Medical Oncology and Radiation Oncology programmes, and this accounts for 2–3% of all medical residents [6].

Participation in a competition for doctoral studies is permitted to people who have a Master’s qualification degree or a higher education degree equivalent to it, the applicants applying to clinical doctorate programs, must have a valid medical practice license. PhD studies are very popular at Lithuanian universities also in medicine field. Only 12% of doctorates are in the medical field. In 2013, there were 195 medical doctorates in Lithuanian universities. In comparison to other European universities, the number of doctorates is way too small, and it would be appropriate to at least double it.

### Educational programme in curriculum

The quality of the programmes as well as the educational and scientific activities of higher education institutions are periodically assessed by the Centre for Quality Assessment in Higher Education. The programme of integrated studies of medicine is a multidisciplinary programme. The workload in credits of studies is 360 European credit transfer system (ECTS) credits. The structure of the study programme is similar to many European medical studies programmes.

In the first three years, medical students undergo preclinical studies: humanitarian–social, fundamental and pre-clinical medicine (e.g., anatomy, human histology and embryology, cytology and parasitology, genetics and movement, respiration, blood circulation, and other models), providing knowledge about the composition of the human body and the functioning of a healthy or a sick person, necessary for further studies. This knowledge and skills are applied and developed in fourth, fifth, and sixth years for solving clinical problems.

The fourth to sixth years of studies are for studying the clinical environment: causes of human diseases, their development mechanisms, features of diseases, their determination modes, and general treatment principles. Particular attention is paid to clinical modules of internal diseases, surgery, intensive medicine, obstetrics–gynaecology, child health, public health, and other modules. The necessary practical skills are acquired in hospital during special medical modules and during the clinical medical practice, which is implemented during the sixth year of studies.

The content of programmes among the universities of Lithuania varies slightly. However this is similar to other countries. According to the ESMO Medical Oncology Status in Europe Survey (MOSES) data, 48% (20/41 countries) have reported that the national situation is heterogeneous, with significant differences in the content and structure of undergraduate teaching between universities in the same country [8].

Oncology in the programmes of medicine is integrated into the teaching courses. As a separate oncology, the only mandatory subject in LUHS is ‘Haematology and Oncology’ (in the fourth year). In VU the mandatory subject is ‘Basics of Clinical Oncology and Cancer Biology’ (in the third year), and an elective subject—‘Cancer prevention’. According to the MOSES report, the teaching of oncology for undergraduate students is foreseen in 85% of the countries, but that of medical oncology in only 50% [7].

### Oncology in other medical programmes

In the universities of Lithuania, there are Bachelor studies (four years) for nursing and public health specialists, Master’s studies (two years) for medical biologists, medical geneticists, medical physicists, nurses, public health and rehabilitation specialists who will encounter oncological patients at their job. In these study programmes, only nurses and medical biologists have oncology as a separate course. Education of the related specialities in oncology is shown in Table 2.

### Residency

The medical/radiation oncology and other medical residencies programmes are described in a recently published article about postgraduate cancer education in Lithuania [6].

### PhD studies

Studies consist of doctoral courses, specific research activities, and the preparation of a doctoral dissertation. PhD courses in universities, related to oncology, are shown in Table 3. Upon completion of the doctoral course, a doctoral thesis must be prepared and publicly defended in order for the candidate to qualify for the doctorate.

### PhD thesis in oncology

**Figure 3.**
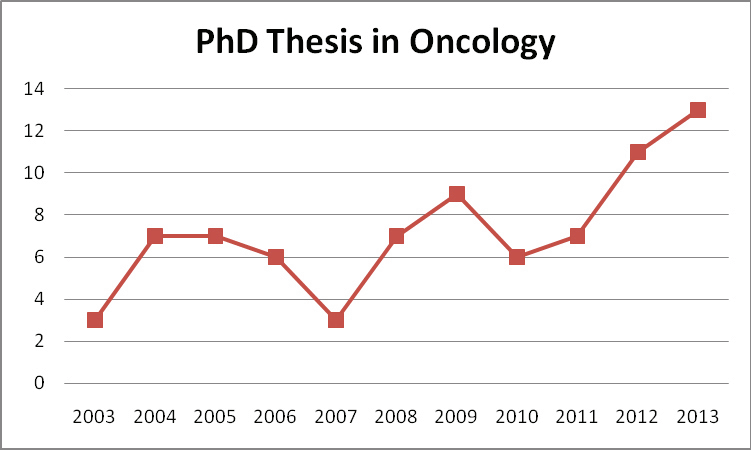
Doctoral thesis in oncology, Lithuania (2003–2013).

During the last decade (2004–2013), 76 dissertations (theses) in the field of oncology where defended at LUHS and VU. Annually, there are about 6–7 such dissertations, and the positive trend is the growth in number of theses in the field of oncology (Figure 3).

**Figure 4.**
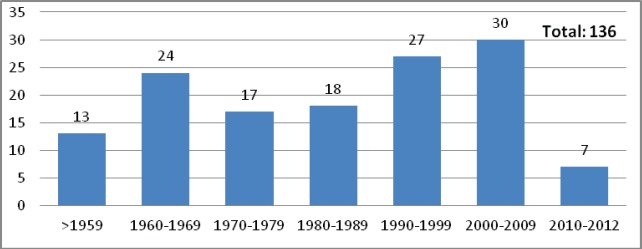
PhD theses defended in National Cancer Institute (1948–2012).

**Figure 5.**
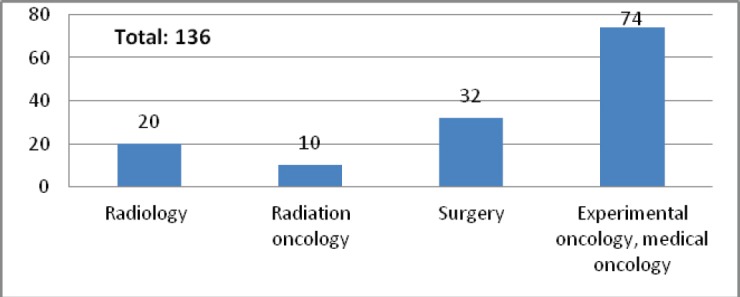
PhD theses defended in National Cancer Institute by speciality (1948–2012).

LUHS performs PhD studies in the field of oncology together with its branches: Clinic of Oncology and Haematology and Laboratory of Oncologic Science; VU performs these studies at branches of medicine faculties together with the National Cancer Institute, where about 30 PhD theses were defended by different speciality doctorants in ten years since 2000 (Figures 4 and 5).

Notably, although the majority of theses in oncology are prepared in the medical field, this subject is also chosen by nursing and public health, biochemistry, and physics doctoral students. This shows that our society has a multidisciplinary approach to cancer and understands the diagnosis and therapy of oncology is not a problem of medicine specialists alone—specialists in other fields of science also actively join in the process.

### Educational resources

The first three years of basic medical education students have their lectures, laboratory work, and practice at the central buildings of the universities. Clinical studies for the upper-year students are carried out at the hospitals. Classes and teaching rooms are equipped with video equipment, computers, and if necessary interactive boards, laboratory equipment, microscopes, negatoscopes, simulators, medical mannequins, etc.

The clinical bases are equipped with the most advanced technology and equipment to diagnose and treat cancer as well as with staff of experts highly skilled in its use: radiologists, pathologists, medical oncologists, surgeons, radiation oncologists, plastic surgeons, and a specially trained support staff.

The medical and radiation oncology residents have mobility through governmental funds that allow the residents to travel abroad every year, visiting the universities or clinics of the European Union (EU), where they can perform certain residency studies in the theoretical and practical aspects of their chosen field.

PhD students also enhance their oncology knowledge through various academic projects and programmes. They are also encouraged to publish their articles at international science magazines, to participate in international events and internships, and to enhance their qualifications while visiting science institutions abroad.

Public funding appropriated for oncology research studies is scarce, which is why joint co-institutional projects and more costly research are subject to the individual initiative of research team leaders and doctorants. This work is based on personal contacts and efforts to raise funds from various foundations supporting activities in the field of science.

### CME providers

Medical professional organisations and societies are the main suppliers and supporters of CME. For example, in 2013 Lithuanian medical societies and associations organised 20 training programmes to increase the qualifications of health specialists, particularly aimed for those in the field of oncology [9].

It is to be noted that oncology field training programmes were intended not only for radiation and medical oncologists, chemical therapists, surgeons, gynaecologists, and haemotologists, but also for nurses, psychologists, psychiatrists, social workers, geneticians, family doctors, and others. The residents are also frequently invited to these types of events.

### Funding for medical education

In Lithuania there are state financed and non financed student tuition. At present, good performing students receive tuition free of charge, while others pay a fee (Table 4).

All residents and PhD students, whose tuition fees are covered, receive a scholarship (Table 5). When a trainee works at a residential clinical base he or she gets a salary: a junior resident (First, second year)–390 EUR/month, a senior resident (third, fourth, and fifth years)–495 EUR/month.

CME expenses are partly covered by state budget funds. The state pays about 60% of fees for specialists’ mandatory qualification courses. The remainder of the amount is covered by another supporting body if appropriate, or by the specialist himself.

Notably, during recent years EU Structural Funds and International Atomic Energy Agency support have contributed to the training of oncologists.

## Conclusions

While newly diagnosed cancer cases continue to increase, the attention to cancer education will become greater and greater.

Lithuanian universities provide basic medical education—integrated studies; postgraduate education–residency studies; and continuing medical education. The basic physician studies will be improved—aiming to bring the future doctor closer to cancer patients. The authors suggest that the undergraduate curriculum should include more oncology-related courses, and the courses of oncology at residential programmes must be extended, in particular for family physicians and internal diseases physicians.

The structure, content, scope, and clinical placements of all studies programmes are appropriate to achieve outcomes sought by the Lithuanian and European legislation requirements. In order to keep pace with the changes in cancer treatment, diagnostic methods, and the implementation of new technologies, the curriculum should be periodically revised.

## Conflicts of interest

The authors declare that they have no conflict of interest.

## Figures and Tables

**Table 1.** Affiliate disciplines of studies for medical residency applicants, 2013.

Residency programme	Affiliate disciplines of studies	
	Graduates of VU	Graduates of LUHS
**Medical oncology**	1. Pathology	1. Forensic Medicine and Clinical Pathology
	2. Pharmacology	2. Haematology and Oncology
	3. Basics of Clinical Oncology	3. Public Health
**Radiation oncology**	1. Pathology	1. Forensic Medicine and Clinical Pathology
	2. Basics of Clinical Oncology	2. Haematology and Oncology

**Table 2.** Education of the related specialities in oncology.

Qualification	Teaching body	Length of programme	Comments
**Radiation technician**	College	Three years	Studies only besides university. Graduates of these studies obtain professional bachelorship in radiology and the qualification of radiology technician.
**Medical Physicist**	University	Two years Master’s degree + Two years training in clinic	Scheme for career medical physicists in Lithuania:
			1. Basic education in physics, biomedicine or technology–four years (Bachelor’s degree) in different universities faculties.
			2. Postgraduate education. Level of Master’s on medical physics–two years (two Lithuania universities are preparing medical physicists).
			3. Postgraduate training in clinical environment (under the supervision of an experienced medical physicist)–two years.
**Nurse**	College	Three years	
	University	Four years Bachelor’s degree	Course ‘Oncology nursing and clinical practice’ (60 hours) in LUHS.
		Two years Master’s degree	Course ‘Oncology and nursing’ (64 hours) in VU.

**Table 3.** PhD courses in universities, related to oncology.

Courses	
**VU**	**LUHS**
Onco-urology	Surgical oncology
Modern radiological diagnostic of tumours	Prognostic and prediction genetic factors of variable tumour localisations
Brain tumours	Medical physics
Chemotherapy and radiotherapy	Tumour related blood diseases
Paediatric haematology and oncology	Tumour biology, new methods in diagnostic and therapy, biotherapy
Tumour etiology, pathogenesis, risk factors an prophylactic	Skin cancer: diagnosis and treatment
Cancer biology and nanomedicine	Oncology
Biomedical statistics (mandatory to all doctors)	

**Table 4.** Charges for basic medical education.

	Vilnius University	Lithuanian University of Health Sciences
Annual fee	2910 EUR/year (1–6 year)	2940 EUR/year (1–4 year) 3930 EUR/year (5–6 year)
Total integrated medical studies	17,460 EUR	19, 620 EUR
Tuition fee for international study programmes	8520 EUR/year Total 51,120 EUR	8300 EUR/year Total 49,800 EUR

**Table 5.** Residents and PhD student scholarships in Lithuania.

	Scholarship amount
Residents	360 EUR/month
First year PhD students	313 EUR/month
Second, third, fourth years PhD students	360 EUR /month

## References

[1] Lithuanian Cancer Registry http://www.vuoi.lt/index.php?-1413089819.

[2] Health Information Centre Institute of Hygiene Vilnius (2013). Health care institutions performance indicators in 2001–2012.

[3] National Institute for Health Development of Estonia the Centre for Disease Prevention and Control of Latvia and Health Information Centre Institute of Hygiene Vilnius (2014). Health in the Baltic Countries 2012.

[4] Lithuanian University of Health Sciences Medical Academy Kaunas (2013). The self-evaluation report 2013 Biomedical sciences, study field medicine, programme of integrated studies medicine.

[5] European specifications, for Basic and Postgraduate Medical Education and Continuing Professional Development (2007). WFME Global Standards for Quality Improvement in Medical Education.

[6] Samalavicius NE (2014). Postgraduate cancer education and training in Lithuania: harmonization according to the EU rules. J Cancer Edu, in press.

[7] Lithuanian Department of Statistics http://db1.stat.gov.lt.

[8] European Parliament (2008). The ESMO MOSES III Survey on the Status of Medical Oncology. http://www.esmo.org/Policy/Recognition-and-Status-of-Medical-Oncology/Status-of-Medical-Oncology-in-Europe.

[9] Ministry of Health of the Republic of Lithuania http://www.sam.lt/go.php/Sveikatos_sistemos_specialistai487.

